# Educating healthcare professionals to improve AF-CARE: lessons from the ESC/EHRA STEEER-AF trial educational programme

**DOI:** 10.1093/europace/euaf142

**Published:** 2025-07-09

**Authors:** Colinda van Deutekom, Karina V Bunting, Tom J R De Potter, Isabelle C Van Gelder, Dipak Kotecha, STEEER-AF education leads, Thompson Robinson, Tatjana Potpara, Eloi Marijon, Pascal Defaye, Pierre Baudinaud, Simon Kochhaeuser, Ursula Rauch, Moritz F Sinner, Marco Proietti, Igor Diemberger, Vincenzo Russo, Stanislaw Tubek, Piotr Buchta, Pawel Balsam, Eusebio García-Izquierdo, Ivo Roca-Luque, Jose M Guerra, Dewi Thomas, Afzal Sohaib, Mark J Davies, STEEER-AF trial steering committee, Giuseppe Boriani, Serge Boveda, Chris P Gale, Eduard Guasch, Samir Mehta, Lluís Mont, Kim Rajappan, Philipp Sommer, Maciej Sterliński

**Affiliations:** Department of Cardiology, University of Groningen, University Medical Centre Groningen, P.O. Box 30.001, Groningen 9700 RB, The Netherlands; Department of Cardiovascular Science, University of Birmingham, Birmingham, UK; NIHR Birmingham Biomedical Research Centre, University Hospitals Birmingham NHS Foundation Trust, Birmingham, UK; Cardiovascular Center, Aalst, Belgium; Department of Cardiology, University of Groningen, University Medical Centre Groningen, P.O. Box 30.001, Groningen 9700 RB, The Netherlands; Department of Cardiovascular Science, University of Birmingham, Birmingham, UK; NIHR Birmingham Biomedical Research Centre, University Hospitals Birmingham NHS Foundation Trust, Birmingham, UK; Julius Center, University Medical Center Utrecht, Utrecht, The Netherlands

**Keywords:** Atrial fibrillation, Stroke prevention, Rhythm control, Education

## Introduction

The European Society of Cardiology (ESC) 2024 atrial fibrillation (AF) guidelines are designed to assist healthcare professionals to better manage their patients and improve AF-CARE.^1,2^ Guideline-adherent treatment is associated with improved prognosis, however implementation of guidelines and adherence to recommendations are complex issues, often with poor performance.^3,4^ The application of guideline recommendations is a major challenge and can lead to undertreatment of patients due to gaps in the knowledge of healthcare professionals.^5–9^ To address these issues, the ESC, the European Heart Rhythm Association (EHRA), and the ESC Council on Stroke designed an educational programme for healthcare professionals on class I and III recommendations on stroke prevention and rhythm control in patients with AF. This was evaluated in the *S*troke prevention and rhythm control *T*reatment: *E*valuation of an *E*ducational programme of the *E*uropean society of cardiology in a cluster-*R*andomised trial in patients with *A*trial *F*ibrillation (STEEER-AF trial).^10^ The aim of this study is to evaluate the STEEER-AF educational programme.

## Methods

STEEER-AF was a cluster randomized clinical trial with 1732 participants across 70 centres in six European countries.^3,10^ The primary objective was to test whether a comprehensive and structured educational programme for healthcare professionals could improve guideline-adherent care for individual patients, focused on stroke prevention and rhythm control of AF. The STEEER-AF educational programme combined country-specific online learning and educational resources along with group and individual support by a local expert trainer. This study focused on the healthcare professionals in centres randomized to the intervention arm that received the educational programme.

A survey was developed by the STEEER-AF trial steering committee to evaluate the educational intervention and provide data for future iteration and deployment. The survey included 28 questions covering learner demographics, overall experience, stroke prevention content, rhythm control content, value of expert trainers, and course usability. The survey was hosted on the SurveyMonkey platform, opened in December 2022 and closed in January 2024. A link to the survey was sent by email to all 195 investigators who took part in the educational programme. At the start of the survey, investigators were asked to provide their consent to participate. Only data from consenting investigators were included in the analysis.

Data from multiple choice and Likert scale questions were presented as counts and percentages, calculated from the number of respondents that answered the question, excluding missing data. Free-text responses were analysed using a thematic approach.

## Results

Seventy survey responses were received from 195 investigators (36% response rate). Two survey responses were excluded (one investigator withheld consent; one duplicate) resulting in 68 survey responses for the final data analysis. Respondents had a wide age range, with 11 (16.4%) aged <30 years, 22 (32.8%) aged 30–39, 15 (22.4%) aged 40–49, and 19 (28.4%) aged ≥50 years. Thirty-eight (56.7%) were women, and professions included medical doctors (58; 89.2%) and nurses (7; 10.8%). There were broad speciality interests, including Primary Care (10; 14.9%), Emergency Medicine (2; 3.0%), Internal Medicine (12; 17.9%), Cardiology (24; 35.8%), and Electrophysiology (19; 28.4%).

### Overall experience

**Figure 1:**
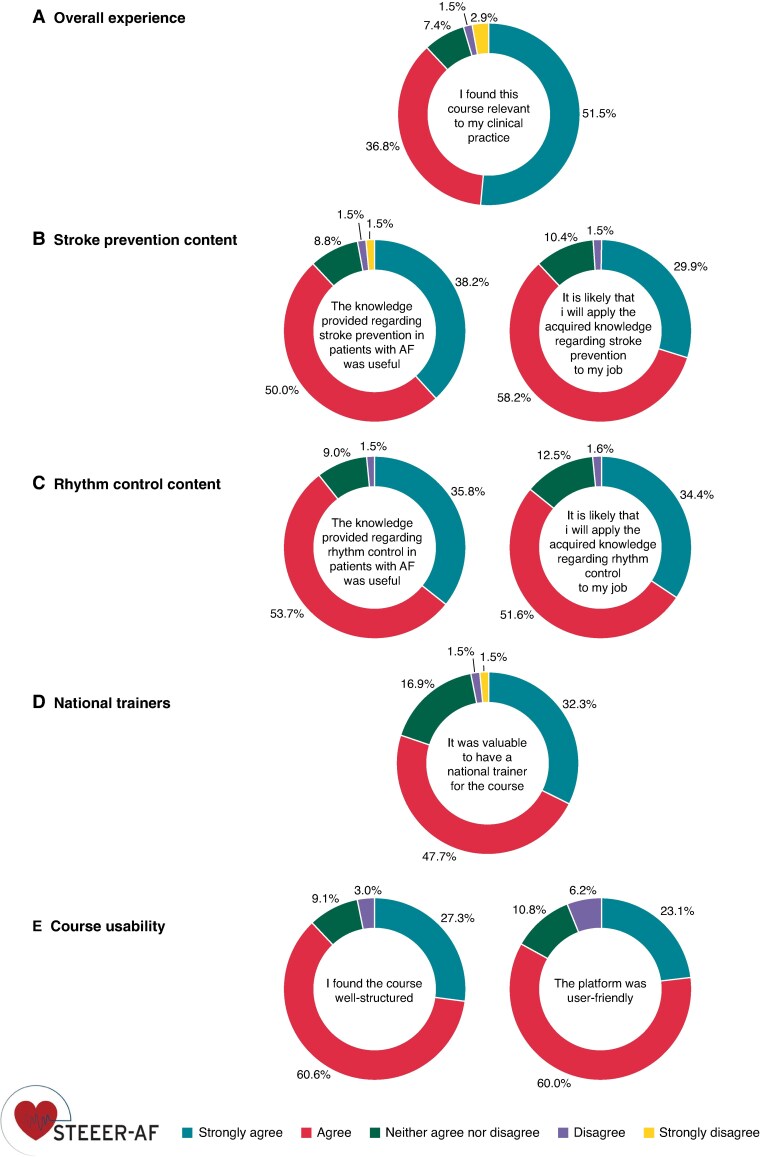
Overview of response distribution to statements regarding the course.

Overall satisfaction and likelihood to recommend this course to a colleague were graded 8 out of 10. Sixty (88.2%) respondents agreed or strongly agreed that the course was relevant to their clinical practice (*Figure 1A*). The main reported strengths were utility and the format of learning; key impacts included improved knowledge on AF management and importance of patient involvement. The main limitation was available time to complete the course.

### Stroke prevention content

Sixty (88.2%) respondents agreed or strongly agreed that the knowledge provided by the course regarding stroke prevention was useful (*Figure 1B*). Fifty-nine (88.1%) respondents agreed or strongly agreed that it is likely that they will apply the acquired knowledge to their job, with key useful aspects being anticoagulation advice and the use of risk scores.

### Rhythm control content

Sixty (89.5%) respondents agreed or strongly agreed that the knowledge provided by the course regarding rhythm control was useful (*Figure 1C*). Fifty-five (86.0%) respondents agreed or strongly agreed that it is likely that they will apply the acquired knowledge to their job, with key useful aspects being different therapeutic options and their indications.

### Expert trainers

Fifty-two (80.0%) respondents agreed or strongly agreed that expert trainers were valuable (*Figure 1D*), the main strengths being the availability to discuss questions, cases, and/or feedback in their native language. Other strengths were their knowledge on local implementation, with value perceived to depend on the communication skills of the trainer.

### Course usability

Fifty-eight (87.9%) respondents agreed or strongly agreed that the course was well-structured (*Figure 1E*). The content was regarded as complete and relevant, but could be improved by reducing module length, adding course-specific videos and personalizing online activity. Fifty-four (83.1%) participants agreed or strongly agreed that the platform was user-friendly and intuitive to use, although technical access could affect the user experience. Learners spent a median of 9.2 h (interquartile range 6.4–13.4) on the platform; regarding time investment, 36 (55.0%) indicated that it was about right, 28 (43.0%) thought it took too much time, and only 1 (2.0%) too little time.

## Discussion

The STEEER-AF randomized clinical trial identified that targeted education for a wide range of healthcare professionals has the potential to affect patient-level treatments for AF where guideline adherence is poor.^10^ In this study, we confirmed general positivity about the educational programme developed by the ESC and EHRA, however the modest response rate may limit representativeness and should be considered when interpreting the findings.

The vast majority of respondents considered the content to be relevant and useful for daily clinical practice. The format of learning, the online platform, and support from local experts were other reported strengths. We also identified limitations and obstructions to learning, including the balance of time vs. clinical commitments. These findings will enhance iteration and deployment of the educational programme beyond the clinical trial. Tailoring education to the individual needs of a healthcare professional has the potential to further enhance efficiency, address new knowledge gaps, improve guideline implementation, and ultimately lead to better and healthier lives for patients with AF.

## Data Availability

The data underlying this article will be shared on reasonable request to the corresponding author.
